# Commentary: Gut microbiome–mediated bile acid metabolism regulates liver cancer via NKT cells

**DOI:** 10.3389/fimmu.2019.00282

**Published:** 2019-02-20

**Authors:** Baolei Jia

**Affiliations:** School of Bioengineering, Qilu University of Technology (Shandong Academy of Sciences), Jinan, China

**Keywords:** gut microbiome, bile acid, liver cancer, NKT cells, immune regulation

A study by Ma et al. (1) showed that gut microbiome composition in mice closely associates with liver cancer by influencing the immune system. This group provided evidence showing that changing commensal gut bacteria in mice affected the accumulation of hepatic CXCR6^+^ natural killer T (NKT) cells through mediation of CXCL16 expression in liver sinusoidal endothelial cells. CXCL16 is the only ligand for the chemokine receptor CXCR6, which mediates NKT cell survival and accumulation in the liver (2, 3). The accumulation of CXCR6 in hepatic NKT cells enhances the production of interferon-γ upon antigen stimulation, which contributes to the inhibition of tumor growth. The accumulation of NKT cells is known to be mainly regulated by a type of *Clostridium* species that metabolizes primary bile acids to secondary bile acids because depletion of *Clostridium* by vancomycin increases hepatic NKT cells and colonization of *C. scindens* induces a rapid decrease in liver NKT cells (1). This evidence highlighted the significant contribution of the gut microbiome to regulating anti-tumor immunity in liver and hepatic cancers.

Human microbiota plays a critical role in maintaining metabolic and immune homeostasis and protecting the host against pathogens (4, 5). The gut microbiota provides a prominent benefit to the host; however, there is also increasing evidence of the involvement of the gut microbiota in human disease (6). The liver is closely linked to the gut because of its anatomical connection via the portal vein. The liver is the first system to acquire nutrient-rich blood via a portal vein from the gastrointestinal tract. Accordingly, the liver is also the first target of metabolites from the gut microbiota, including bile acids, choline, short-chain fatty acids, indole derivatives, and lipopolysaccharides (7). Bile acids can be classified into primary bile acids and secondary bile acids, which are synthesized by the liver and by bacterial metabolism in the colon, respectively. Recently, emerging evidence has also indicated direct associations between obesity, gut microbiota, secondary bile acids, and hepatocellular carcinoma (HCC) (8, 9). Dietary obesity induces a clear expansion of gram-positive gut microbiota, especially *Clostridium* clusters XI and XVIa, in mice with a high-fat diet (8, 9). The elevation in the strains increased the levels of deoxycholic acid (DCA), a secondary bile acid, and lipoteichoic acid (LTA), a major cell wall component in gram-positive bacteria. The accumulation of the two molecules in the livers of HFD mice treated by chemical carcinogen cooperatively enhanced the Toll-like receptor 2 (TLR2)-mediated signals by the upregulation of the receptor, which induced overexpression of cyclooxygenase2 (COX2), catalyzing the production of prostaglandin E_2_ (PGE_2_). Accumulation of PGE_2_ suppressed anti-tumor immunity through a PTGER4 receptor on CD8 cells, thereby contributing to HCC progression (9).

Compared with previous studies, this study contributes to advances in the related field in the following ways. First, the results from Ma et al. (1) indicated that altering the gut microbiome caused the accumulation of both CD8 cells and NKT cells; however, depleting CD8 cells alone had minor effects on the tumor inhibition caused by elimination of commensal gut bacteria, and antibiotic treatment of tumor-bearing mice lacking NKT cells did not reduce liver tumor size. These results suggest that NKT cells are critical for effects on hepatic tumor growth induced by alterations in the gut microbiome. Second, Ma et al. (1) also provided evidence that increasing primary bile acids increased hepatic NKT cells and enhanced tumor inhibition but that increasing secondary bile acids had opposing effects. These analyses ascertained the beneficial effect of primary bile acids functioning as a regulator to enhance tumor inhibition. These findings indicate an axis of bile acids and CXCL16, CXCR6, and NKT cells that regulate live cancer. Third, DCA, a secondary bile acid, has always been speculated to be a promoter of liver cancer (10). This study showed that other secondary bile acids also played important roles. For example, ω-muricholic acid (ω-MCA) but not DCA decreased *cxcl16* mRNA expression. Increasing ω-MCA expression by feeding was shown to inhibit the activation of liver sinusoidal endothelial cells. These findings present new knowledge of the function of different secondary bile acids.

The findings from Ma et al. shed light on the prevention and treatment of liver cancer by targeting the gut microbiota in clinical application. The data directly indicated that elimination of gram-positive bacteria by vancomycin from the gut prevents tumorigenesis (1). The data from the study also solidified the evidences of influence of liver health by diets, probiotics, and antibiotics, which affect the composition of the human gut microbiota. This research cautioned that *Clostridium* colonization in gut promotes tumor growth, on the other hand, the commensal *Bifidobacterium* can enhance antitumor immunity and regulate the therapy efficacy by blocking programmed cell death 1 ligand 1 (PD-L1) (11). Because there is individual variability in response to diets, endobiotics, and xenobiotics (12), the studies of precision editing of the gut microbiota are needed to prevent live cancer. Furthermore, the results from Ma et al. also raised the questions on the influence of gut microbiota on the monoclonal antibodies therapies by PD-L1 or PD-1 (programmed cell death 1) blockade. Nivolumab, an anti-PD-1 monoclonal antibody, has been approved by the FDA for liver cancer in 2017 (13). Recent studies showed that gut microbiomes modulate the efficacy of immunotherapies against melanoma and epithelial tumors (14, 15). On the basis of this study, further studies should be performed to assess the effect of gut microbiomes on the immunotherapies to cure liver cancer in clinical trials.

This study provided a comprehensive analysis of the relationship among the gut microbiome, the immune system, and liver cancer. However, this research invokes three related questions. First, the mechanism of bile acids regulating *Cxcl16* expression is still unclear. CXCL16 is a small cytokine with a C-X-C motif with an O-glycosylated mucin-like stalk, a transmembrane helix and a cytoplasmic domain with a potential tyrosine phosphorylation site. These features allow CXCL16 to be expressed as a soluble chemokine as well as a cell surface-bound molecule (16). Further analysis should be performed to elicit if CXCL16 bind bile acids directly or through other molecules. Second, which secondary bile acids did contribute significantly to liver cancer? It has been reported that DCA induced liver cancer and nodules in rats in 1991 (17). Yoshimoto and Loo further showed that DCA was one of the factors facilitating liver cancer development (8, 9). The current research indicated that ω-MCA should be one of the critical players to promote liver cancer. ωMCA is a transformed from primary bile acid βMCA by three strains in a cooperative way, including one *Eubacterium lentum* strain and two *Fusobacterium sp*. strains (18). While DCA is transformed from cholic acid by *Clostridium* clusters XI and XVIa. Then it is critical to elucidate the contributions of different bacteria and secondary bile acids to promote live cancer. Third, can the findings be applied to humans? Approximately 1% of hepatic lymphocytes are NKT cells in humans; however, the cells constitute up to 40% of hepatic lymphocytes in mice. Promisingly, it was shown that primary bile acid CDCA levels in human samples were correlated with *CXCL16* expression, whereas secondary bile acid glycolithocholate (GLCA) levels were inversely correlated. Furthermore, mucosal-associated invariant T (MAIT) cells, which are prevalent in human liver, can also express CXCR6 that can bind CXCL16. This evidence suggests that the current study could be translated into clinical practice. cancer. However, comprehensive analysis of human liver tissue is necessary for clinical application considering the differences between humans and mice.

## Author Contributions

BJ wrote the commentary.

### Conflict of Interest Statement

The author declares that the research was conducted in the absence of any commercial or financial relationships that could be construed as a potential conflict of interest.
